# Mechanical small bowel obstruction secondary to acute appendicitis

**DOI:** 10.1093/jscr/rjaf1075

**Published:** 2026-01-16

**Authors:** Muhi D Barazi, Malek Zanbrakji, Ahmad Safra, Ahmed Hegab, Maseer Bade, Mohammed Bayasi

**Affiliations:** Department of Biosciences, Rice University, 6100 Main Street, Houston, Texas 77005, United States; Department of Health Policy, Georgetown University, 3700 O Street, NW Washington, DC 20057, United States; Department of Surgery, INOVA Health System, 3300 Gallows Road, Falls Church, Virginia 22042, United States; Department of Surgery, INOVA Health System, 3300 Gallows Road, Falls Church, Virginia 22042, United States; Department of Surgery, INOVA Health System, 3300 Gallows Road, Falls Church, Virginia 22042, United States; Virginia Surgery Group, Private Practitioner, 1850 Town Center Parkway, Reston, Virginia 20190, United States

**Keywords:** small bowel obstruction, appendicitis, appendicular band syndrome, appendicular tie syndrome, appendico-ileal knot, case report

## Abstract

Mechanical small bowel obstruction (SBO) secondary to acute appendicitis is rare, described only in isolated reports. Herein we present a photo-documented case illustrating this mechanism. A 39-year-old female presented postoperative day 6 from laparoscopic myomectomy with periumbilical abdominal pain and distention. Computed tomography showed possible SBO, and after 24 h without improvement, exploratory laparoscopy was performed revealing a necrotic-appearing appendix completely encircling and obstructing the small bowel proximal to the terminal ileum. Appendectomy was performed facilitating patient recovery. Acute appendicitis can cause SBO through several mechanisms. Mechanical obstruction by an inflamed appendix is a rare etiology that’s been variably called appendicular knot, appendico-ileal knot, appendicular band syndrome, or appendicular tie syndrome. This may be associated with closed-loop obstruction and bowel strangulation warranting prompt surgery. Although rare, acute appendicitis should be considered as a possible cause of SBO of unknown etiology.

## Introduction

Appendicitis is a common cause of an acute abdomen leading to emergency surgery [1]. Small bowel obstruction (SBO) is also common, implicated in up to 16% of annual surgical admissions in the United States [2]. However, mechanical obstruction of the small bowel due to acute appendicitis is extremely rare [1]. It’s often challenging to diagnose on the basis of clinical findings and imaging and may be discovered intraoperatively. Herein, we report a photo-documented case of mechanical SBO due to an acutely inflamed appendix encircling the small bowel.

## Case report

A 39-year-old female presented to the hospital complaining of sharp, periumbilical abdominal pain beginning several hours earlier. She further relayed chills, bloating, and vomiting. She had a history of uncomplicated laparoscopic myomectomy 6 days prior with a normal postoperative course. She reported a bowel movement after symptom onset and a normal bowel movement at presentation. She denied other pertinent past medical or surgical history.

**Figure 1:**
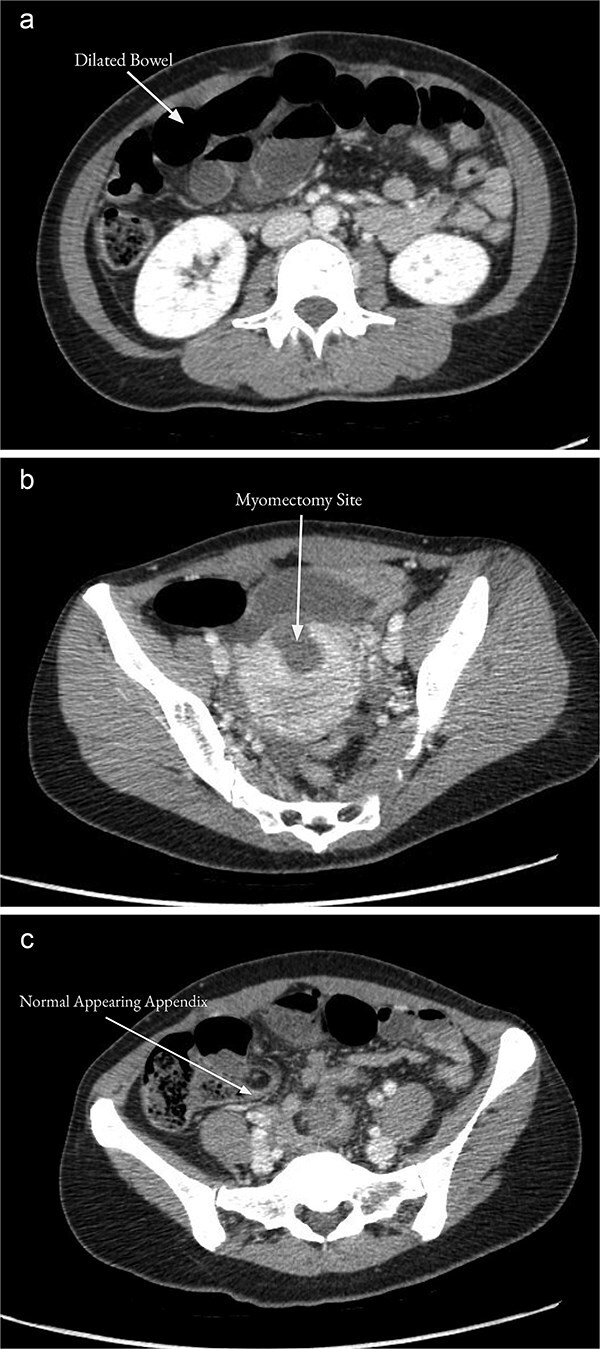
CT of the abdomen and pelvis with contrast on the day of admission showed multiple distended mid-distal small bowel loops indicating a possible small bowel obstruction (a). There was focal low density along the uterine myometrium consistent with the history of recent myomectomy (b). The appendix appeared normal (c).

On examination, the patient was alert, oriented, and appeared uncomfortable but in no acute distress. Her abdomen was soft but distended with diffuse palpation tenderness. There was no rebound tenderness or guarding. Her supraumbilical incision was clean, dry, and intact. Laboratory workup was unremarkable, including a normal white blood cell count (WBC, 9.6 × 10^3^/μL), hemoglobin (13.2 g/dL), and hematocrit (40.1%). Computed tomography (CT) of the abdomen and pelvis with contrast demonstrated multiple distended mid-distal small bowel loops with a transition to decompressed bowel in the right lower quadrant, consistent with a possible mid-small bowel obstruction (Fig. 1a). There was a small hemoperitoneum and focal low density along the uterine myometrium consistent with the history of myomectomy (Fig. 1b), and a normal-appearing appendix (Fig. 1c).

**Figure 2:**
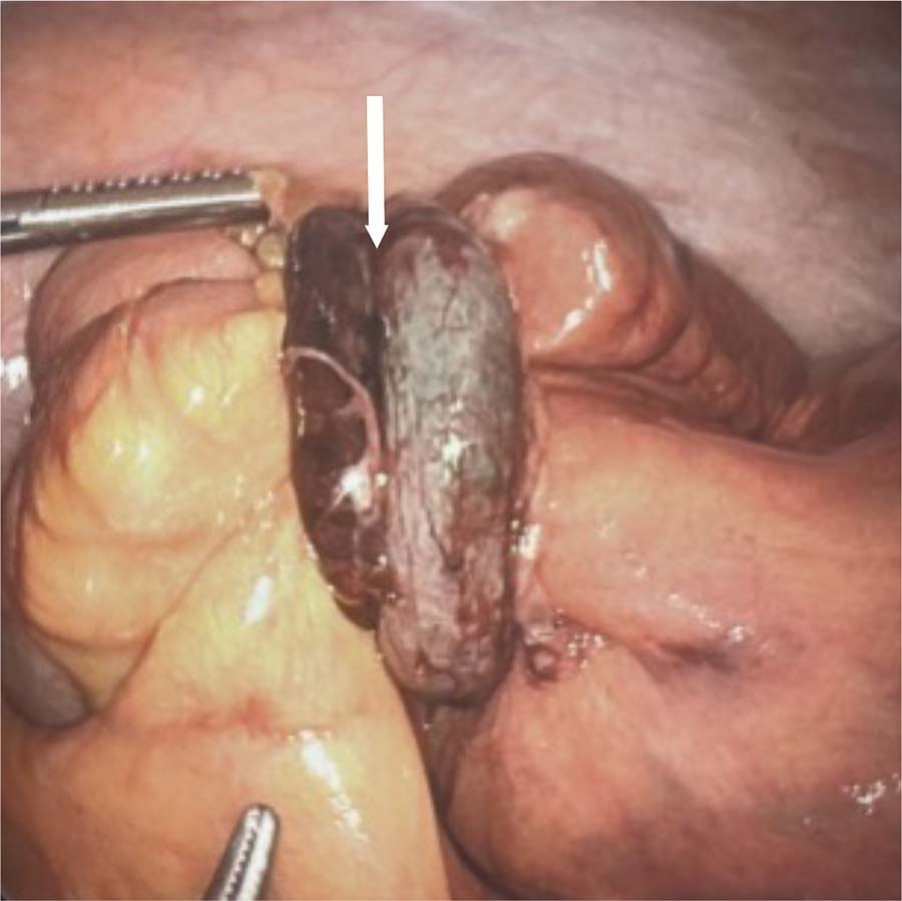
Intraoperative photograph of the necrotic-appearing appendix (arrow) encircling the small bowel 10 cm proximal to the terminal ileum causing a mechanical small bowel obstruction with a clear transition point.

The patient was admitted for suspected SBO versus postoperative ileus and underwent nasogastric tube placement. A water-soluble contrast challenge was performed, after which the patient developed nausea, vomiting, and worsening abdominal pain. On the following day, there was no return of bowel function, no improvement in the patient’s symptoms, and WBC had increased to 12.1 × 10^3^ /μL, so diagnostic laparoscopy was performed. Intraoperatively, dilated but viable small bowel was noted, and a necrotic appearing appendix was discovered encircling the small bowel 10 cm proximal to the terminal ileum. This was causing frank mechanical SBO with a clear transition point (Fig. 2). The appendix was resected with pathology revealing acute hemorrhagic appendicitis. The patient tolerated the procedure and was discharged home on postoperative day 2 after return of bowel function and oral intake.

## Discussion

SBO secondary to appendicitis can occur via several different mechanisms [3, 4]. In adynamic obstruction, which is the most common, appendicular inflammation spreading to the adjacent small bowel, cecum, or posterior peritoneum leads to paralytic ileus [1, 4]. Mesenteric ischemia is a rare mechanism, where the inflamed appendix becomes adherent to the mesentery adjacent to the ileocolic artery, causing thrombosis, and ischemia of the terminal ileum [4]. Finally, our case demonstrates a mechanical SBO secondary to acute appendicitis, which is an atypical cause in the literature [1, 4–10].

In mechanical SBO due to appendicitis, the small bowel becomes compressed by the inflamed appendix [4]. The appendix is a mobile organ, creating risk for it to adhere to structures when inflamed [4]. Its tip may adhere to the cecum, retroperitoneum, mesentery, or ileum, forming a space through which a loop of bowel can herniate [7]. A closed-loop obstruction and strangulation of the herniated loop can ensue [4]. Alternately, the appendix may wrap around a single tract of bowel to form a ‘tourniquet,’ as in our case [5]. These findings typically occur with a closed-loop obstruction, and have been variably described as appendicular knot, appendico-ileal knot, appendicular band syndrome, or appendicular tie syndrome [4, 7, 8]. However, these terms are not well defined nor consistently used throughout the few reported cases. Although there was no strangulation or closed-loop obstruction in our case, it’s similar to the previously described cases of appendicular band syndrome in that the appendix formed a ring around a segment of small bowel to create a mechanical obstruction. Compression of the appendix itself or the small bowel can lead to ischemia, necrosis, and perforation; therefore, prompt surgical treatment of this condition is necessary [10].

The diagnosis of appendicitis-associated mechanical SBO can be challenging. Patients may present with either a predominant clinical picture of appendicitis, or, as in our case, primarily that of SBO [4]. While CT often shows a characteristic transition between dilated and normal bowel, the causative appendicitis may be obscured and not easily seen on imaging [4, 5]. Therefore, when SBO signs and symptoms predominate, appendicitis is often an intraoperative finding, as in our case, where the appendix appeared normal on CT imaging. Our patient’s recent surgical history was also a confounding factor as a postoperative ileus was considered; however, her recent myomectomy was ultimately unrelated to the pathology found. Treatment is excision of the inflamed appendix releasing the obstruction, and may be approached with laparoscopy or laparotomy as appropriate [5]. However, if necrosis of the small bowel has occurred, resection, and anastomosis of the bowel may be necessary [5].

## Conclusion

This case demonstrates that although rare, mechanical SBO secondary to appendicitis can occur, and should be considered in the differential diagnosis for SBO of unknown etiology [4].
